# Laying the groundwork for a culturally sensitive pediatric primary care eating disorders intervention for the Latino community: insights from focus groups

**DOI:** 10.1186/s40337-025-01451-3

**Published:** 2025-11-28

**Authors:** Isabel Rodriguez, Mariana Valdez-Aguilar, Mae Lynn Reyes-Rodríguez, Shelby Ortiz, Cynthia M. Bulik, Emily M. Pisetsky

**Affiliations:** 1 School of Medicine, University of North Carolina at Chapel Hill, Chapel Hill, NC USA; 2 Department of Psychiatry, University of North Carolina at Chapel Hill, Chapel Hill, NC USA; 3 Department of Psychiatry, University of Michigan, Ann Arbor, MI USA; 4 Department of Nutrition, University of North Carolina at Chapel Hill, Chapel Hill, NC USA; 5 Department of Medical Epidemiology and Biostatistics, Karolinska Institutet, Stockholm, Sweden

**Keywords:** Eating disorders, Disordered eating, Adolescents, Primary care, Cultural adaptation, Latino, Hispanic, Early intervention, Qualitative research

## Abstract

**Background:**

Adolescents and caregivers from the Latino community face significant barriers to accessing treatment for eating disorders (ED) and disordered eating (DE) including cultural stigma, language barriers, and limited availability of culturally congruent care. Most existing ED/DE interventions were developed for non-Hispanic White populations, often limiting their application to families from the Latino community. To address this disparity, we are developing First Approach Skills Training- *Trastornos de la Conducta Alimentaria* (FAST-TCA), a culturally adapted version of FAST-DE, a brief workbook-based intervention for ED/DE designed for implementation in pediatric primary care.

**Methods:**

This study used a community-engaged iterative process to inform the first steps of the cultural adaptation of FAST-DE into FAST-TCA. Two rounds of semi-structured focus groups were conducted with each of two stakeholder groups: Latino caregivers of adolescents with ED/DE histories and Latino adolescents with lived experience of ED/DE. Focus groups were recorded, transcribed, and analyzed using a codebook thematic analysis informed by a phenomenological perspective.

**Results:**

Seven themes emerged from caregiver focus groups: psychological and social influences on ED/DE development, treatment experiences, family context, barriers to care, treatment priorities, caregiver empowerment, and workbook design and accessibility. Four themes were extracted from adolescent focus groups: navigating cultural identity and ED/DE, family dynamics and support, the need for sensitive and thoughtful content delivery, and ED/DE recovery experience reflections. Across both groups, participants emphasized the importance of accessible, culturally congruent, family-centered care and offered specific feedback that informed the revisions to the FAST-TCA workbook, including the incorporation of culturally relevant foods and expanded caregiver psychoeducation.

**Conclusions:**

Focus group insights guided the development of the first draft of FAST-TCA, an initial cultural adaptation of FAST-DE. This study demonstrates the utility of community-engaged methods in enhancing the cultural relevance of ED/DE interventions. The next phase of the cultural adaptation will occur following the pilot implementation of FAST-TCA, where additional feedback from adolescents and caregivers will inform further refinement of the intervention.

## Abstract (plain language summary)

Eating disorders (ED) and disordered eating (DE) are increasingly common in adolescents, but specialty care remains hard to access. To improve access to care, we are developing First Approach Skills Training- Disordered Eating (FAST-DE), a brief ED/DE intervention. FAST-DE is a workbook-based program that consists of five sessions with adolescents with ED/DE, their caregivers, and a generalist mental health provider, designed to provide early support and reduce the need for specialty treatment.

In this study, we began the cultural adaptation of FAST-DE into First Approach Skills Training-*Trastornos de la Conducta Alimentaria* (TCA) to meet the needs of families from the Latino community. We conducted focus groups with adolescents and caregivers from the Latino community who had lived experience with ED/DE. Participants shared their ED/DE treatment experiences and provided input on the FAST-TCA intervention. Feedback from both groups was used to draft the first version of the workbook and create additional caregiver education materials.

## Background

Primary care settings have witnessed a concerning increase in prevalence and severity of eating disorders in adolescents [1]. This has led to the health care system becoming overwhelmed, resulting in long wait lists and delayed care [2]. Although primary care providers (PCPs) frequently detect and diagnose adolescent patients with EDs [3], many feel inadequately prepared to treat EDs and refer those patients to specialty clinics [3]. To improve access to early ED intervention, we are developing First Approach Skills Training-Disordered Eating (FAST-DE)—a workbook-based primary care intervention for adolescents with EDs and disordered eating (DE), aligned with the widely used FAST interventions [4–6]. In this paper, we report on findings from the focus groups used to inform the development of FAST– *Trastornos de la Conducta Alimentaria* (TCA), a culturally adapted version of FAST-DE designed to meet the needs of the Latino community.

FAST-DE builds on the broader FAST framework, which has been successfully implemented for other mental health conditions including depression, anxiety, and trauma [4, 5]. These brief behavioral interventions were designed to improve access to mental health care by providing structured, evidence-based treatment via primary care settings, where mental health concerns are often first identified. In this model, patients are identified and referred by PCPs to generalist mental health providers, embedded within the primary care setting, with the goal of reducing barriers to care and addressing needs early. FAST-DE is a workbook-based intervention that consists of five sessions, each lasting 30 to 45 min. Each session involves the adolescent patient, their caregiver(s), and a generalist mental health provider who guides them through the workbook. The goal of FAST-DE is to equip adolescents and their families with evidence-based strategies to address DE behaviors early, potentially preventing the progression to more severe EDs and reducing reliance on specialty care.

Early intervention is critical to improving prognosis for ED/DE; however, specialist treatment is inaccessible to many, especially those in underserved communities, highlighting the need for interventions such as FAST-DE [6–8]. The Latino community, one of the fastest growing ethnic groups in the United States of America (USA) [9], faces significant barriers to care including limited access to culturally congruent providers, stigma, financial constraints, and mistrust of the health care system [7, 8, 10, 11]. People from the Latino community are less likely to both seek and receive specialized ED/DE care than their White counterparts [7, 12, 13]. For instance, Marques et al. found significant differences in mental health service utilization across racial and ethnic groups for those with a history of bulimia nervosa (54.07% among the Latino community versus 77.35% among Whites) and binge-eating disorder (42.83% Latino community versus 78.89% Whites) [14]. This is concerning because binge-purge type disorders are the most prevalent in the Latino community [15]. Given these disparities, primary care settings may serve as a more accessible and trusted environment for receiving ED/DE care, as has been shown for other mental health conditions [16]. 

Addressing the disparities in ED/DE care experienced by the Latino community requires interventions that are congruent with their cultural values, which can be achieved through cultural adaptation. Most existing ED treatments have been developed by and for predominantly non-Hispanic White populations, limiting their applicability across diverse cultural contexts [17, 18]. To our knowledge, only three studies have been published documenting cultural adaptation for the Latino community, all of which have been for Latinas with binge-purge type eating disorders [19–21]. Those adaptations primarily targeted individuals meeting criteria for an ED based on the Diagnostic and Statistical Manual of Mental Disorders and did not include early onset or DE behaviors. The process of cultural adaptation involves modifying existing evidence-based interventions to align with the cultural values, beliefs, and practices of a specific target population, with the goal of increasing the treatment’s relevance and effectiveness while maintaining the core therapeutic components [17]. The main framework guiding this cultural adaptation process is the Ecological Validity Framework developed by Bernal et al. [22] This model identifies eight elements to guide cultural adaptation across phases of the process (language, persons, metaphors, content, concepts, goals, methods, and context). Cultural adaptation was embedded in our intervention by involving bilingual, culturally congruent providers in the development of the English and Spanish versions. Once the Spanish version was finalized and aligned with the eight elements, the next step was gathering feedback from the target population. Engaging community members as stakeholders is vital to this process to ensure that the adaptation truly reflects the needs of the target population [17]. An effective method for facilitating this engagement is the use of focus groups, which provide a structured yet flexible setting for gathering insights from individuals with lived experience.

Building on this framework, the present study was designed to inform the initial cultural adaptation of FAST-TCA by conducting two sets of focus groups with members of the Latino community who had lived experience with ED/DE. One set of focus groups was with caregivers of adolescents with a history of EDs or DE and the other with adolescents with a history of ED/DE (not necessarily related to the caregivers). The focus groups gathered participants’ perspectives on their experiences navigating their ED/DE, including their treatment experience, as well as their feedback on the draft FAST-TCA program and proposed materials. Information from the focus groups was then incorporated into the subsequent version of FAST-TCA. Our iterative and collaborative approach provides a blueprint for adaptations of FAST-DE and other interventions to better meet the needs of diverse cultural groups.

## Methods

### Workbook development (overview)

The FAST-TCA workbook was developed through a structured and iterative process. Initially, a multidisciplinary team of ED providers developed, formatted, and illustrated the FAST-DE workbook, drawing from evidence-based ED interventions, including family-based treatment (FBT) [23] and enhanced cognitive-behavioral therapy (CBT-E) [24]. Throughout the development process, input was incorporated from primary care consultants and the Seattle Children’s FAST team that has developed and implemented multiple FAST interventions in pediatric primary care [4, 24]. This workbook was then translated from English to Spanish and underwent a preliminary cultural adaptation by a team of ED clinicians (IR, MVA, and MLRR) from the Latino community (representing three different Latino subgroups). This process involved reviewing the content for cultural relevance and accessibility, including adapting idiomatic expressions and changing character names to reflect more common Latino and gender-neutral names. An overview of the initial FAST-TCA workbook, consisting of a summary of session topics and examples of planned activities for each session, was presented to a caregiver focus group and an adolescent focus group. The workbook was then further adapted by incorporating feedback obtained from the first round of focus groups. During the second round of focus groups, additional feedback was obtained and incorporated to finalize the workbook. The adaptation process was guided by the Ecological Validity Framework [22]. 

### Flow of study

The study included six steps: (1) develop, illustrate, and format the FAST-DE English workbook; (2) translate the FAST-DE workbook into Spanish and complete a preliminary cultural adaptation to create the first FAST-TCA workbook draft; (3) conduct first round of focus groups to obtain feedback on overall program structure; (4) revise and edit the FAST-TCA workbook-based on feedback from the first round of focus groups; (5) conduct a second round of focus groups and obtain feedback on a revised version of the FAST-TCA workbook; and (6) incorporate feedback from focus groups into a preliminary FAST-TCA workbook that will be pilot-tested.

### Recruitment

Study participants were recruited through flyers distributed at the university, at local Latino clinics, and at an ED treatment center. Participants were also notified via targeted emails and posts through research registries such as ResearchMatch and Research for Me, which allow researchers to contact individuals who have opted in to receive information about relevant studies. Additionally, recruitment messages were shared on social media platforms such as Meta and X. Eligibility criteria for the caregiver focus groups were: (1) caregiver of an adolescent with a history of an ED or DE, (2) able to read and speak Spanish, (3) self-identified as part of the Latino community, and (4) living in the USA (including Puerto Rico). To participate in the adolescent focus groups, participants had to meet the following eligibility criteria: (1) age 15 to 19 years, (2) had a history of an ED or DE, (3) self-identified from the Latino community, and (4) living in the USA including Puerto Rico. Participants were remunerated with $50 gift cards for their participation in each focus group. Study approval was obtained from the UNC Biomedical Ethics Board (#24–0895). Informed consent was obtained from all adult participants and parents/guardians of adolescents, and assent was obtained from all adolescents.

### Focus groups

Two semi-structured virtual focus groups were facilitated for both the caregiver group and the adolescent group by a licensed clinical psychologist, a postdoctoral clinical psychologist, and a registered dietitian/medical student, all of whom are from the Latino community, native Spanish speakers, and experienced in treating EDs. The caregiver focus groups were conducted in Spanish and the adolescent groups were conducted in English, reflecting the language preferences and comfort levels of each group—most caregivers were native Spanish speakers, whereas the adolescents primarily spoke English in their daily lives. Each group lasted approximately two hours.

Prior to the first group, participants received an overview of the proposed FAST-TCA topics to review. This overview included session titles and brief descriptions of topics such as understanding EDs, emotional regulation, body image and self-esteem, managing compensatory behaviors, and relapse prevention. At the beginning of the session, one of the facilitators (a registered dietitian/medical student) gave a brief presentation summarizing this information to guide the discussion and gather initial impressions. Following the presentation, one of the facilitators asked predetermined questions to the group to guide a semi-structured discussion, prompting the group to share their previous experiences with ED/DE and ED treatment. Discussion topics also included general knowledge of ED/DE, experiences seeking treatment, perceptions of the role of health care providers, communication with providers, and any additional reflections they wished to share. We also gathered feedback on the proposed workbook topics—specifically, which felt most relevant, what content might be missing, and how the material could be made more engaging and culturally appropriate. Prior to the second group meeting, participants were sent a more detailed outline of each workbook session, changes to be made based on feedback from the first group, and a revised formatted draft of module one of the workbook, which focuses on psychoeducation related to ED/DE. Additional feedback on the revised materials was gathered. The scripted questions used to guide focus group discussions are included in the supplemental materials.

### Analysis

The focus groups were recorded, transcribed, and checked for accuracy by the facilitators. The data were coded and analyzed in NVivo 14 by two independent coders (IR and MVA) using a codebook thematic analysis approach with a phenomenological perspective, following best practices outlined by Braun and Clarke [25–27]. The analysis process involved familiarization with the data, generation of initial codes, and iterative development and refinement of the themes, codes, and codebook. To mitigate bias, coders independently coded the transcripts multiple times. After each round of independent coding, the coders met to discuss discrepancies, resolved them through consensus, and refined the codebook. A third coder (MLRR) was available to resolve any disagreements; this was only needed twice, as most discrepancies were resolved through discussion between the two primary coders. This iterative process ensured that the themes accurately represented the data and captured the lived experience of the participants. The shared cultural background and professional experience in ED treatment among the coders enhanced the reliability and depth of the analysis.

## Results

### Participants

#### Caregiver groups

**Fig. 1:**
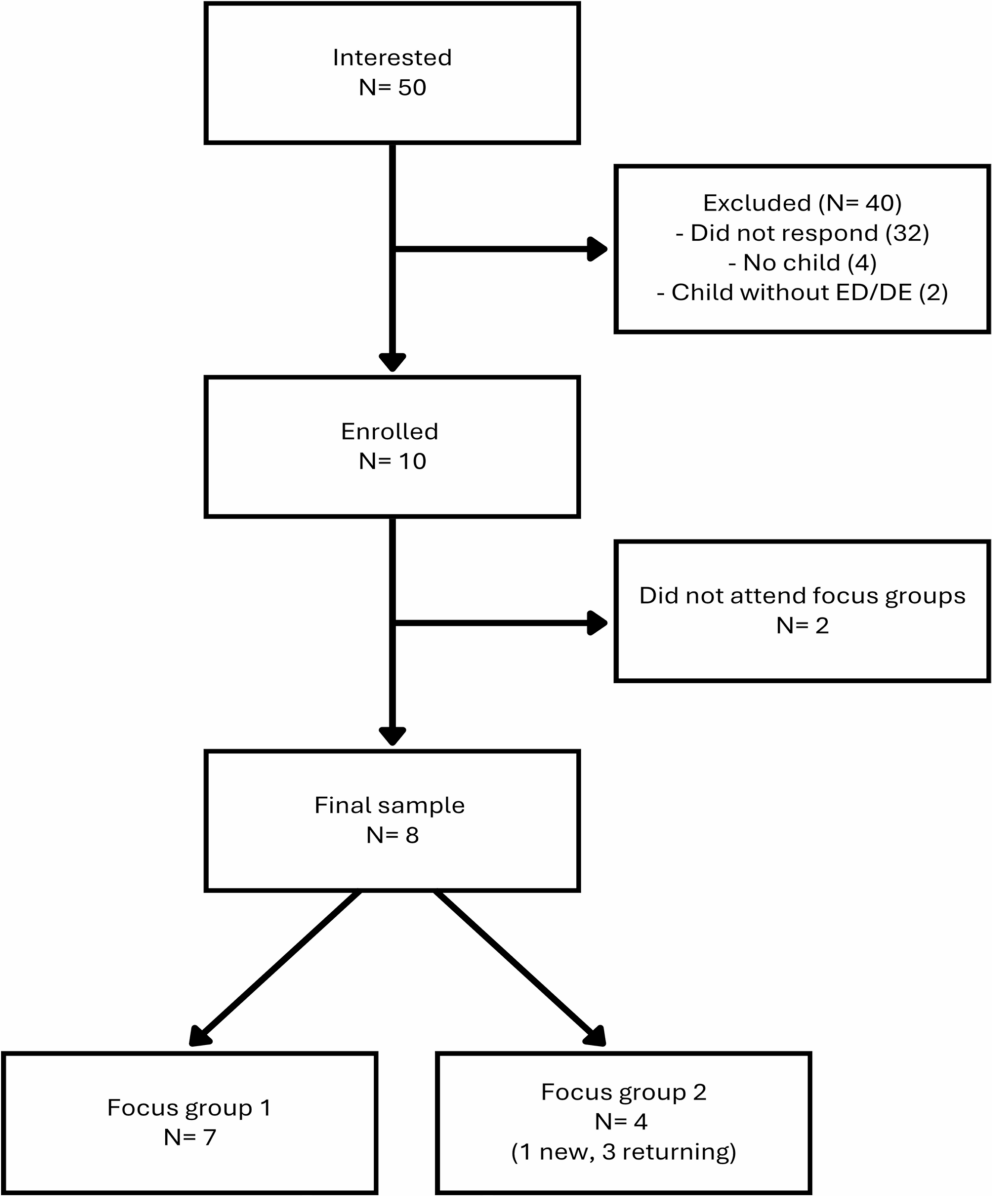
Participant recruitment flow for caregiver focus groups

The final sample included eight participants (Fig. 1). Seven individuals attended the first group, and four attended the second group (one of whom had not attended the first group). Participation in the first group was not required to join the second.

#### Adolescent groups

**Fig. 2:**
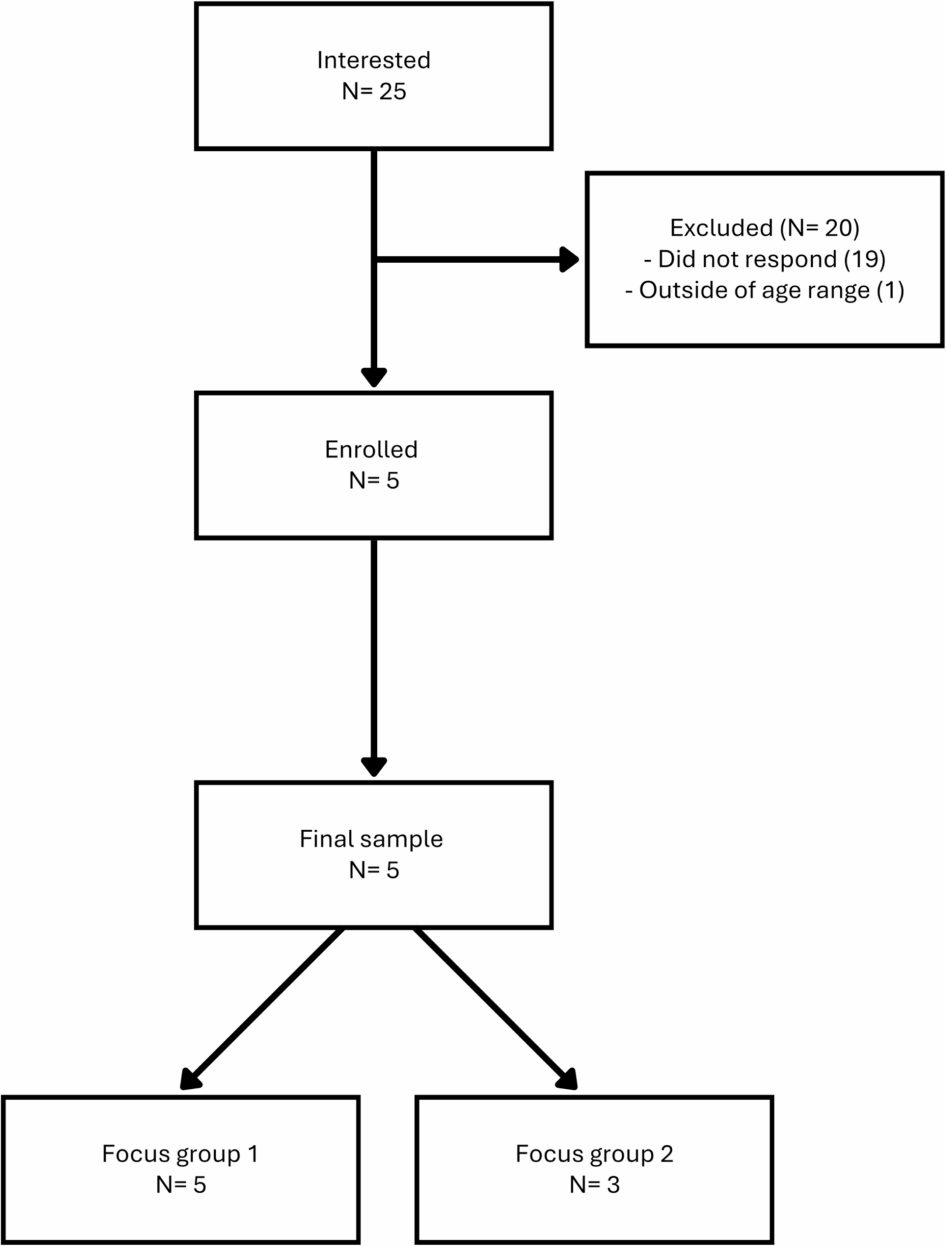
Participant recruitment flow for adolescent focus groups

The final sample included five participants (Fig. 2). Five individuals attended the first group and three attended the second group.

### Focus group findings

#### Caregiver focus groups themes

Seven major themes were identified from the caregiver groups (Table 1). The themes were divided into two broad categories: (1) experiences with ED/DE and (2) feedback on FAST-TCA. We present each theme with illustrative translated quotes (original Spanish versions provided), in Table 1.

**Table 1:** Final codebook for caregiver focus groups

Category	Theme	Code	Definition	Quotes
Experience with ED/DE	Psychological and social influences on the development and maintenance of ED/DE	Impact of social rejection and bullying	Factors related to social exclusion and bullying that contribute to the development and maintenance of ED/DE. This includes experiences of bullying, social isolation, and lack of peer support	Participant (P) 1: “El tema de la apariencia que en el colegio es muy fuerte. Las niñas quieren ser aceptadas, por eso dejan de comer, se visten de otras maneras. Para nosotros puede ser un problema sencillo, pero para ellas es su mundo. Es todo lo que más les importa.” (The issue of appearance is very strong at school. Girls want to be accepted, so they stop eating, they dress differently. For us it might seem like a simple problem, but for them it is their world. It’s all that matters most to them)
		Distorting effects of social media	The distorted effects of social media platforms on body image and eating behaviors. This includes exposure to unrealistic beauty ideals, pressure to meet certain body standards, and constant comparison with other users	
		Need for social acceptance and validation	The intense desire to be accepted and belong to a social group. This can lead to dysfunctional eating behaviors to fit in or be validated by peers	
	Treatment experiences	Caregiver willingness and initiative in seeking help	The likelihood and motivation of caregivers to seek treatment once they recognize an issue with ED/DE. This includes the willingness to acknowledge there is a problem and take steps to address it by seeking professional help	P1: “No fue una experiencia nada agradable, porque los psiquiatras, el trato que tenían con ella era casi como robótico… Yo estaba con ella y la experiencia para mí fue bien desagradable. Mi hija entró con muchas expectativas de que podían ayudarla, pero empezó a sentirse ignorada mientras el proceso pasaba.” (It was not a pleasant experience at all because the psychiatrist treated her almost robotically…I was with her, and the experience for me was very unpleasant. My daughter had many expectations that they could help her, but she began to feel ignored as the process went on) P6: “Creo que es muy importante llegar a la [terapeuta] indicada que tenga como el ‘feeling’ adecuado con el niño para que ellos puedan abrirse y expresar realmente todo lo que tienen que expresar para poder después recibir la ayuda que necesitan.” (I think it’s very important to find the right [therapist] who has the appropriate connection with the child, so they can open up and truly express everything they need to express in order to receive the help they need)
		Perception of treatment effectiveness	Opinions and experiences on how the treatment has been helpful or not in managing the ED/DE. This includes satisfaction with the results and the perception of changes in mental and physical health	
		Quality of relationship with health care provider	The quality of interaction between the patient and the health care provider (physician), including effective communication, empathy, and support received during treatment	
		Developing a strong therapeutic alliance	The collaborative and trusting relationship between the therapist (psychologist) and the patient, essential for treatment success. This includes the patient’s level of comfort with the therapist and the perception of being understood and supported	
	Family context	Caregiver emotional struggles	The emotional difficulties faced by caregivers due to their children’s ED/DE. This includes feelings of worry, responsibility, and stress	P7: “Hay que mantener las emociones para no transmitir el sufrimiento y el dolor que uno pasa como mamá al tener que ver un hijo o una hija sufrir por un trastorno de alimentación.” (You have to manage your emotions to avoid transmitting the suffering and pain you go through as a mother when you see your son or daughter suffer from an eating disorder) P1: “Lo que le ayudó a salir al final fue ella misma y conversando conmigo contándome lo que ya sentía, desfogándose conmigo y buscando ella la manera de afrontar sus temores.” (What ultimately helped her was herself, talking to me, telling me how she felt, venting to me, and finding her own way to face her fears)
		Family support and communication	The way in which caregivers and families provide emotional and practical support to the patient with an ED/DE, as well as how they address and discuss these issues with their children. This includes participation in treatment, fostering a positive environment, and providing resources and assistance	
		Caregiver nutritional perceptions and practices	The practices and perceptions of caregivers regarding the feeding and nutrition of their children. This includes caregivers’ attitudes and beliefs about feeding, nutrition, and their eating habits	
	Barriers to treatment	Cultural and linguistic barriers	Obstacles related to cultural beliefs, values, and norms that hinder access to and effectiveness of treatment. This includes cultural stigmas, lack of understanding about ED/DE, linguistic barriers, and challenges in interacting with health care providers due to cultural differences	P6: “Es difícil…No hay un recurso que de pronto tú puedas usar para llegar a esa persona adecuada para el caso específico. Y muchas veces necesitas ayuda adicional. No sé, que sea bilingüe, que sea… No sé qué aparte lo cubra tu seguro. O sea, como que hay muchas variables extra que también entran en juego. Entonces, como que no hay un recurso realmente que sirva para poder llegar a la persona indicada, la que realmente pueda ayudarle a tu hijo con el problema específico de tu hijo” (It’s difficult…There isn’t a resource that you can suddenly use to find the right person for the specific case. And many times, you need additional help. I don’t know, someone bilingual, someone… I don’t know, that is also covered by your insurance. I mean, there are many extra variables that come into play. So, there’s really no resource that helps you find the right person who can truly help your child with their specific problem)
		Systemic health care challenges	Problems within the health care system that prevent patients from receiving adequate treatment. This can include lack of resources, long waiting lists, limited access to specialists, issues with medical insurance, and the complexity of understanding and navigating the health care system	
		Distrust in the health care system	Lack of trust in the health care system and health care professionals by patients and their families. This can be based on past negative experiences or a general perception of ineffectiveness	
FAST-TCA feedback	Treatment priorities	Customizing session priorities (incorporating flexibility)	Opinions on which topics should be addressed first in FAST-TCA treatment sessions. This includes the importance of certain topics from the participants’ perspective	P4: “A lo mejor para los chicos es algo silencioso, que lo quieren pasar desapercibido. No lo quieren hacer notar, no lo conversan. Los padres nos damos cuenta menos. No lo sé. Pero si ponemos algo de [varones] que también están en esta situación de incomodidad con su imagen corporal podemos hacer que más jóvenes varones asuman que les puede pasar.” (Maybe for boys, it’s something silent that they want to go unnoticed. They don’t want to make it known, they don’t talk about it. Parents notice it less. I don’t know…But if we include something about [boys] that are also in this situation of experiencing discomfort with their body image, we can make more young males realize that it can happen to them too) P5: “Incluir las comidas Latinas hace que se sienta más común, más Latino, más hacia uno, más hacia nuestra cultura.” (Including Latino foods makes it feel more familiar, more Latino, more aligned with us, more with our culture) P3: “Hay que entregarle herramientas al joven o a la joven para que pueda enfrentar estos [desafíos]. Yo pienso en las redes sociales. Lo primero que se me viene a la mente es como las adolescentes ven las redes sociales y se sienten presionadas por llegar a esos estereotipos inalcanzables. Entonces, a lo mejor ayudarles a entregarles herramientas a la familia y al paciente para que pueda distinguir entre lo que es real y no real…” (We need to provide young people with tools to face these [challenges]. I think about social media. The first thing that comes to mind is how teenagers see social media and feel pressured to achieve those unattainable stereotypes. So, maybe helping to give tools to the family and the patient to distinguish between what is real and what is not…)
		Gender inclusivity and representation of males	Consideration and representation of the unique needs and experiences of men in the treatment of ED/DE. This includes adapting therapeutic approaches to address gender differences, including examples and materials that reflect gender diversity, and promoting a treatment environment that recognizes and validates male experiences	
		Importance of emotional factors	The importance of including emotional factors in the treatment of ED/DE. This includes how emotions and emotional regulation influence eating habits and body image	
		Incorporation of Latino foods	Integration of traditional foods and culinary practices from Latino culture in the treatment of ED/DE	
		Managing external influences	Strategies for managing the influence of school, social media, and extracurricular activities on adolescents’ eating behavior and body image. This includes techniques for coping with academic pressure, peer interactions, and the healthy use of social media, distinguishing online reality from fiction (i.e., photo editing on social media)	
	Empowering caregivers for effective support	Psychoeducation tools and resources for caregivers	The need for educational resources and strategies designed to help caregivers better understand ED/DE and how they can effectively support their children. This includes exercises, guides, and strategies	P1: “Si yo conozco más lo que le está pasando a mi hija y la puedo comprender mejor entonces mi trato con ella y la manera como yo me comporto también va a mejorar. Pasamos por estrés. Pasamos noches sin dormir, pensando en cómo vamos a ayudarlos en solucionar este problema. También es un problema grande para nosotros, entonces comprenderlo y que nos den herramientas para nosotros lidiar con ellos [sería útil].” (If I know more about what is happening to my daughter and can understand her better, then my interactions with her and my behavior will also improve. We go through stress. We spend sleepless nights thinking about how we are going to help them solve this problem. It is also a big problem for us, so understanding it and being given tools to cope with it [would be helpful])
		Active caregiver involvement	The importance of active caregiver participation in the treatment and recovery process in FAST-TCA. This includes their role in implementing strategies and providing ongoing emotional support	
	Workbook design and accessibility	Simple formatting with examples	The importance of a clear and understandable structure in the workbook, using accessible and direct language. This includes providing examples of the activities to ensure the instructions are clear	P2: “Las imágenes y las visualizaciones son bastante importantes. Yo soy muy visual y aprendo más rápido y me gusta leer, pero muy poco…Por eso la visualización creo que va con todos. Si eres lector o no eres lector, la visualización va con todo.” (Images and visualizations are quite important. I am very visual and learn more quickly that way, and although I like to read, I don’t read much…I believe visualization works for everyone. Whether you are a reader or not, visualization goes with everything)
		Visual and interactive elements	Inclusion of visual elements such as images and videos, designed to capture attention, facilitate learning, and make the process more dynamic and engaging. This includes suggestions to add images and graphics, and comments on current images	

### Category 1: Experiences with ED/DE

#### Theme 1: Psychological and social influences on the development and maintenance of ED/DE

All of the caregivers spoke about the influence of various social and psychological factors as drivers of the child’s ED/DE, emphasizing the impact of social rejection, the need for social acceptance, and the effects of social media as significant contributors. They discussed how these factors influence body image and self-esteem.

Participant (P) 1: “The issue of appearance is very strong at school. Girls want to be accepted, so they stop eating, they dress differently. For us it might seem like a simple problem, but for them it is their world. It’s all that matters most to them.”

#### Theme 2: Treatment experiences

Most caregivers mentioned negative treatment experiences, particularly with physicians. Most emphasized the importance of their child developing a strong therapeutic alliance with their therapist.

P1: “It was not a pleasant experience at all because the psychiatrist treated her almost robotically…I was with her, and the experience for me was very unpleasant. My daughter had many expectations that they could help her, but she began to feel ignored as the process went on.”

#### Theme 3: Family context

Caregivers frequently highlighted the role of family support in the treatment process and the emotional impact of the ED/DE on the family as a whole. They discussed how their own beliefs and practices around nutrition influenced their child’s eating behaviors.

P7: “You have to manage your emotions to avoid transmitting the suffering and pain you go through as a mother when you see your son or daughter suffer from an eating disorder.”

#### Theme 4: Barriers to treatment

Several treatment barriers were identified, including cultural and language barriers, systemic health care challenges, and distrust in the health care system by both caregivers and their children. Participants mentioned feeling overwhelmed by the health care system and noted that finding effective treatment required many variables that were out of their control to align correctly.

P6: “It’s difficult…There isn’t a resource that you can suddenly use to find the right person for the specific case. And many times, you need additional help. I don’t know, someone bilingual, someone… I don’t know, that is also covered by your insurance. I mean, there are many extra variables that come into play. So, there’s really no resource that helps you find the right person who can truly help your child with their specific problem.”

### Category 2: FAST-TCA feedback

#### Theme 5: Treatment priorities

Caregivers shared their priorities for the workbook content. They underscored the need to include representation of ED/DE in males, the incorporation of traditional Latino foods, addressing emotional factors of ED/DE, and strategies for managing external influences such as school and social media.

P5: “Including Latino foods makes it feel more familiar, more Latino, more aligned with us, more with our culture.”

P3: “We need to provide young people with tools to face these [challenges]. I think about social media. The first thing that comes to mind is how teenagers see social media and feel pressured to achieve those unattainable stereotypes. So, maybe helping to give tools to the family and the patient to distinguish between what is real and what is not…”.

#### Theme 6: Empowering caregivers for effective support

Many caregivers emphasized the importance of their involvement in the treatment program and the need for educational tools to better understand their child’s condition.

P1: “If I know more about what is happening to my daughter and can understand her better, then my interactions with her and my behavior will also improve. We go through stress. We spend sleepless nights thinking about how we are going to help them solve this problem. It is also a big problem for us, so understanding it and being given tools to cope with it [would be helpful].”

#### Theme 7: Workbook design and accessibility

Caregivers provided feedback on the design and accessibility of the workbook. They emphasized the importance of a clear, simple format with practical examples and interactive images to engage both caregivers and adolescents.

P2: “Images and visualizations are quite important. I am very visual and learn more quickly that way, and although I like to read, I don’t read much…I believe visualization works for everyone. Whether you are a reader or not, visualization goes with everything.”

### Adolescent focus groups themes

Four major themes were identified within the two focus group transcripts for the adolescent groups (Table 2).

**Table 2:** Final codebook for adolescent focus groups

Theme	Code	Definition	Quotes
Navigating cultural identity and ED/DE	Influence of cultural expectations	Cultural norms and values within the Latino community that shape attitudes toward body image, food, beauty standards, and other factors that influence the development and maintenance of ED/DE. This includes the impact of gender roles, family expectations, and societal pressures specific to the Latino community	P2: “For me, it was more of my extended family who was definitely pushing the cultural expectations of like therapy is bad [and psychiatric] medicines are bad. I grew up religious so a lot of times I was just told to pray which is totally okay, but for me, praying would not instantly make me better.” P3: “I’m Christian and so my mom was able to book a Christian therapist… It was really helpful to have that aspect since my faith played a big role in my healing process.”
	Stigma of mental health and treatment	Attitudes, beliefs, and behaviors in the Latino community that contribute to the stigma surrounding mental health and seeking treatment. This includes the perception that mental health struggles are a sign of personal weakness or failure, and the fear of shame or judgment within the community	
	Role of religion and spirituality	The influence of religion and spiritual beliefs in shaping the understanding of ED/DE and approaches to recovery. This includes how adolescents from the Latino community and their families may turn to faith-based solutions, seeking support through prayer, religious guidance, or spiritual practices as part of their coping strategies and healing process	
	Unique needs of the Latino community	The importance of culturally specific considerations for adolescents from the Latino community in ED/DE treatment. This includes the need for care that incorporates their values, traditions, language, and family dynamics, along with the recognition of the lack of culturally congruent resources currently available for the community	
Family dynamics and support	Family misunderstanding of ED/DE	Lack of awareness or misconceptions within the family about the nature and seriousness of ED/DE. This includes minimization of symptoms and viewing the disorder as a phase	*P2*: “There was a lot of resentment…and a lot of feeling unheard because [my caregiver] chose to only focus on the food rather than the mental and emotional reasoning behind [the disorder].” *P5*: “I’ve noticed that there isn’t as much of an emphasis and just general education for Hispanic families about eating disorders and topics concerning mental health, because it’s often just overlooked, at least in my household and my family. So, it’s really important that programs like these offer more education to the parents on [these] topics.”
	Need for family education	The expressed need for programs and materials that educate family members about ED/DE. This includes information on recognizing symptoms, understanding the emotional and psychological aspects of the disorder, and how to provide effective support during recovery	
	Perception of family support and engagement in treatment	Adolescents’ perceptions of family support during treatment and recovery. This includes whether they felt their families were engaged, provided emotional support, and participated in treatment efforts. It also explores how family engagement, or the absence of it, influenced their recovery experience	
Thoughtful content delivery with sensitive framing	Presenting the emotional complexity of ED/DE	Recognizing and presenting ED/DE as complex mental illnesses that go beyond weight or body image concerns. This includes understanding that ED/DE can be rooted in emotional distress, trauma, and psychological pain, with behaviors often serving as maladaptive coping mechanisms	P2: “I am always worried about reading [a list of] symptoms because for some people it can feel really good like, ‘Oh, I finally have an explanation of why I feel this way’, and for other people it can be…almost like a checklist of like ‘Oh, I haven’t had all these symptoms…so therefore I’m not sick enough. I don’t deserve treatment.’” P3: “I definitely think body acceptance is where I’m at. I don’t think I will ever fully love my body for like how it looks, but I’ve accepted [it for] what it can do and what it serves me for. So that’s a great way to approach it [in the workbook].”
	Use of objective and neutral language	The importance of using language that does not reinforce disordered behaviors and remains neutral and factual. This includes being mindful of wording around weight, food, and body image, while avoiding language that may trigger emotional distress or promote harmful behaviors	
	Flexible approaches to body image	Exploring approaches to body image that respect diverse perspectives, from self-love, which encourages positive regard toward one’s body, to body neutrality, which focuses on valuing the body’s function and health without pressure to love its appearance. This includes recognizing that individuals may connect with these perspectives differently, based on their body image journey and self-acceptance	
	Crisis resources and support	The importance of offering resources to individuals experiencing a crisis related to ED/DE, including referrals to emergency mental health services, hotlines, treatment centers, and other forms of immediate intervention	
	Encouraging open, honest, and respectful dialogue	The importance of fostering a therapeutic environment where caregivers and adolescents communicate openly and respectfully with each other and with the provider. This includes creating a space where individuals feel comfortable being truthful, without fear of judgment or consequences, and are encouraged to express their thoughts and concerns honestly	
ED/DE recovery experience	Coming to terms with having an ED/DE	The process by which adolescents come to understand and make sense of their ED/DE. This includes initial reactions to the diagnosis, implications for their identity and lifestyle, and reflecting on how the diagnosis shapes their perception of health	P2: “For a long time I knew what I had, but I didn’t accept what I had. It wasn’t until I was hospitalized that I was like, ‘Okay I have to be serious.’” P3: “I was just in a really dark place in my life but going therapy really healed that in me…it was really helpful for me.”
	Negative experiences in treatment	Adverse experiences adolescents encountered during treatment for their ED/DE. This includes dissatisfaction with overall care, unhelpful interactions with staff, feeling misunderstood or invalidated in treatment settings, and concerns about how treatment content was delivered	
	Relationship with health care provider	Adolescents’ perception of the quality of their relationship with their primary health care provider. This includes feelings of trust, comfort, and being understood, as well as how these factors influenced their engagement with their provider	
	Denial of illness	Experiences in which adolescents denied or minimized aspects of their illness or were unaware of harmful behaviors. This includes reluctance to acknowledge the severity of their ED/DE, believing they have control over the disorder, viewing symptoms as normal, or rejecting the need for treatment/professional help	

#### Theme 1: Navigating cultural identity and ED/DE

Adolescents discussed how their identification with the Latino community, including cultural norms, social expectations, religious beliefs, and family values shaped their views on body image, food, and mental health. They noted stigma surrounding mental health, including family skepticism about therapy and psychiatric medications. Many highlighted the need for ED/DE treatment approaches that integrate cultural considerations from the Latino community to effectively address their lived experiences.

P2: “For me, it was more of my extended family who was definitely pushing the cultural expectations of like therapy is bad [and psychiatric] medicines are bad. I grew up religious so a lot of times I was just told to pray which is totally okay, but for me, praying would not instantly make me better.”

#### Theme 2: Family dynamics and support

Many adolescents shared that family members often lacked a comprehensive understanding of ED/DE, highlighting the need for educational resources. They noted caregivers tended to focus on the food-related aspects of the disorder, often overlooking the emotional and psychological components. Adolescents described varying levels of familial support and engagement in treatment, which influenced their recovery experience.

P5: “I’ve noticed that there isn’t as much of an emphasis and just general education for Hispanic families about eating disorders and topics concerning mental health, because it’s often just overlooked, at least in my household and my family. So, it’s really important that programs like these offer more education to the parents on [these] topics.”

#### Theme 3: Thoughtful content delivery with sensitive framing

Adolescents emphasized the importance of presenting workbook content in a way that highlights the emotional complexity of ED/DE, avoids reinforcing harmful behaviors, and promotes an open dialogue between caregivers and adolescents. They highlighted their preferences for the use of neutral, objective language and flexible approaches to body image.

P2: “I am always worried about reading [a list of] symptoms because for some people it can feel really good like, ‘Oh, I finally have an explanation of why I feel this way’, and for other people it can be…almost like a checklist of like ‘Oh, I haven’t had all these symptoms…so therefore I’m not sick enough. I don’t deserve treatment.’”.

#### Theme 4: ED/DE recovery experience

Adolescents reflected on their experiences with ED/DE from diagnosis to treatment. They discussed how the diagnosis impacted their identity, shared negative treatment experiences, and highlighted the importance of a trusting relationship with their primary health care providers.

P2: “For a long time I knew what I had, but I didn’t accept what I had. It wasn’t until I was hospitalized that I was like, ‘Okay I have to be serious.’”.

## Discussion

The findings from the caregiver and adolescent focus groups provide important insights into the experiences, challenges, and needs surrounding ED/DE treatment within the Latino community and underscore the importance of systematic cultural adaptation processes to ensure cultural congruence of any ED/DE treatment. Through a community-engaged approach, this study contributed to the development of FAST-TCA, a culturally adapted version of FAST-DE that reflects the lived experiences and priorities of adolescents and caregivers from the Latino community. Importantly, this paper offers an initial approach and community informed process for adapting ED/DE interventions that could be used in other cultural contexts.

Consistent with prior research, caregivers highlighted a range of systemic and sociocultural barriers to effective treatment including stigma, lack of culturally concordant providers, and difficulty navigating the health care system [8, 28]. Adolescents further underscored the role of cultural identity in shaping their experiences with ED/DE. Their reflections suggested a sense of cultural “in-betweenness”, navigating mental health challenges within the context of both USA norms and traditional Latino family values. This common experience, particularly for second-generation immigrants, has been referred to as bicultural stress—the psychological strain that arises from managing competing cultural norms, values, and expectations [29]. 

A key finding that emerged from both groups was the need for improved education for caregivers. Caregivers expressed a strong desire to support their adolescents and often focused on food-related behaviors to do so. Adolescents echoed this, noting that they felt the emotional and psychological aspects of their illness were overlooked. This disconnect may further reflect cultural and generational differences in conceptualizing mental health. First-generation immigrant caregivers often demonstrate high levels of resilience, which can serve as a protective factor [29, 30]. Additionally, they face structural challenges in establishing themselves in a new country, such as securing stable employment, housing, and legal documentation, and caring for their family, that may require prioritizing immediate, tangible needs [31]. In contrast, their second-generation children may have more stability in these areas and as a result, more space to engage with psychological concerns. This generational framing offers a possible explanation for the caregiver-adolescent disconnect seen in treatment priorities and highlights the value of integrating greater psychoeducation for caregivers into culturally adapted ED/DE interventions.

The cultural adaptation was guided by the Ecological Validity Framework and informed by the focus group findings to ensure cultural congruence in the intervention design (Tables 3 and 4). Based on the focus group findings, our team developed caregiver psychoeducation materials for FAST-TCA that extend beyond those included in FAST-DE. The materials help caregivers better understand ED/DE and provide them with tangible, actionable strategies to support their adolescents. Additional adaptations to enhance the cultural relevance and accessibility of FAST-TCA included revisions to character names and storylines, incorporation of culturally familiar foods, clarification of activity formats, and the option for inclusion of extended family members in the intervention (Table 4).

**Table 3:** Application of the ecological validity framework to FAST-TCA adaptation

Ecological Validity Framework element	Corresponding adaptations in FAST-TCA	Rationale
Language	Workbook was adapted in English and translated to Spanish so it is available in both languages depending on family and individual acculturation level. Spanglish is incorporated throughout English version	Ensures linguistic accessibility and reflects the bilingual communication patterns common in families from the Latino community living in the USA
Person	Facilitators and providers matched by language and cultural background	Promotes trust and therapeutic alliance by increasing cultural and linguistic concordance
Metaphors	Integration of Latino foods, family meal examples, and culturally resonant sayings	Leverages familiar cultural metaphors to enhance engagement and relatability
Content	Incorporation of familism, shared meals, and family-centered psychoeducation	Emphasizes family connection as both a cultural value and a therapeutic mechanism, while expanding psychoeducation to equip caregivers with practical strategies to support adolescents
Goals	Shared emphasis on children’s well-being as a cultural value guiding both therapists and caregivers	Reinforces alignment between therapeutic aims and culturally held priorities around child health and family cohesion
Methods	Use of storytelling and illustrative vignettes	Reflects preferred narrative forms of communication common in Latino culture
Context	Implementation in pediatric primary care and delivered via telehealth	Uses trusted, lower stigma care settings viewed as safe by families, with telehealth offering additional comfort amid immigration-related concerns

**Table 4:** Examples of cultural adaptations from FAST-DE to FAST-TCA

Original FAST-DE content	Revised FAST-TCA content	Rationale	Relevant theme (subtheme)- caregiver groups	Relevanttheme (subtheme)-adolescent groups
Character name: Jamie	Character name: Ari	While Jamie is typically a male name in Latino culture, Ari serves as a gender-neutral nickname resonant in both Spanish- and English-speaking families from the Latino community	Treatment priorities (Gender inclusivity and representation of males)	
Jamie’s story focused on eating behaviors	Ari’s story expanded to include emotional experiences of ED/DE	Reflects adolescent feedback emphasizing the emotional aspects of ED/DE	Treatment priorities (Importance of emotional factors)	Thoughtful content delivery with sensitive framing (Presenting the emotional complexity of ED/DE)
Example entrée: Spaghetti with meatballs	Example entrée: Chicken quesadilla	More culturally familiar foods	Treatment priorities (Incorporation of Latino foods)	Navigating cultural identity and ED/DE (Unique needs of the Latino community)
Activity with open-ended questions for caregivers	Activity with checkboxes for caregivers, with optional space for elaboration	Simplifies activity and increases accessibility	Workbook design and accessibility (Simple formatting with examples)	
No section on social media	Added section on *Replacing Unhelpful Social Media*	Addresses adolescent concerns about harmful online influences and equips families with practical tools	Treatment priorities (Managing external influences)	
No additional caregiver resources	Caregiver handouts for each session	Provides supplemental psychoeducation and strategies for caregivers	Empowering caregivers for effective support (Psychoeducation tools and resources for caregivers)	Family dynamics and support (Need for family education)
Caregivers and adolescents attend sessions	Caregivers, adolescents, and other family members may attend sessions	Acknowledges the importance of extended family involvement in households from the Latino community	Empowering caregivers for effective support (Active caregiver involvement)	Family dynamics and support (Need for family education)

This study had several strengths. The use of a community-engaged, iterative process ensured that the adaptation of FAST-TCA was grounded in the lived experiences and cultural contexts of adolescents and caregivers from the Latino community. The focus groups also included individuals from four Latin American countries, providing diverse representation within the Latino community. This breadth is essential because the Latino community is not a monolith; this community has varied backgrounds and cultures, which influence their perspectives and needs. By incorporating perspectives from both caregivers and adolescents, we integrated multiple stakeholder perspectives, allowing for the identification of shared concerns as well as points of divergence. The caregiver group included caregivers of both boys and girls with a wide range of ED/DE presentations, whereas the adolescent group included a range of genders, allowing for a broad representation of experiences. Although formal diagnostic data were intentionally not collected, participants voluntarily described experiences consistent with anorexia nervosa, bulimia nervosa, binge eating behaviors, restrictive eating related to texture sensitivities, and body image concerns, highlighting the heterogeneity of presentations represented. Cultural and linguistic concordance between facilitators and participants helped build rapport, creating a comfortable environment for participants to share their insights openly.

A limitation of the study was the small sample size and the limited number of focus groups conducted, raising the possibility that thematic saturation was not achieved. Despite the small sample, several consistent themes emerged across caregiver and adolescent groups, supporting the validity of the findings. Another limitation was that fewer participants attended the second focus group in both the caregiver and adolescent groups, which may have affected the breadth of feedback on the treatment materials presented during those sessions. Despite these limitations, the study provides valuable insights into the cultural adaptation of ED/DE treatment for the Latino community.

## Conclusions

This study represents an important first step in the cultural adaptation of FAST-DE for use with Latino families. Through focus groups, we identified key cultural, structural, and psychological factors that influence treatment engagement and outcomes in this population. Findings underscore the urgent need for culturally congruent ED/DE interventions that address the specific values, beliefs, and barriers experienced by Latino families.

The next phase will pilot the initial draft of FAST-TCA in pediatric primary care, followed by qualitative interviews with adolescents and caregivers to guide further refinement. This iterative process offers a scalable model for adapting ED/DE interventions to better serve diverse and underserved populations.

## Data Availability

The datasets used and/or analyzed during the current study are available from the corresponding author on reasonable request.

## Abbreviations

ED: Eating disorder
DE: Disordered eating
FAST-DE: First approach skills training- disordered eating
FAST-TCA: First approach skills training-Trastornos de la Conducta Alimentaria
USA: United States of America
